# Listen, Empower, Co-Create: Adapting Boot Camp Translation Methods to Create Culturally Responsive Health Messages and Materials

**DOI:** 10.3928/24748307-20250612-01

**Published:** 2025-07

**Authors:** Jamie H. Thompson, Jennifer S. Rivelli, Priyanka Gautom, Gloria D. Coronado

**Affiliations:** a Kaiser Permanente Center for Health Research, Portland, Oregon; b University of Arizona Cancer Center and; c Mel and Enid Zuckerman College of Public Health, University of Arizona, Roy P. Drachman Hall, Tucson, Arizona.

## Abstract

**Background:**

Community engagement is key to developing culturally responsive public health interventions that resonate with diverse populations and promote health equity.

**Brief Description of Activity:**

We applied an adapted version of Boot Camp Translation (BCT), a community-based participatory approach, to develop culturally and locally relevant messaging and materials for diverse populations. This adapted BCT approach focuses on three core themes: (1) Listen, (2) Empower, and (3) Co-Create, or LEC. The LEC method helps community leaders and champions learn from community members about barriers and gaps in health care (listen), share health information in a collaborative way (empower), and develop messages and materials that resonate with and motivate community members to take control of their own health (co-create).

**Implementation:**

LEC follows a 3 to 4 month process: an in-person session with expert presentations and group discussions, followed by two virtual meetings to share and refine co-created messages and materials.

**Results:**

The LEC approach was successfully conducted in diverse communities, engaging participants in preferred venues like churches, clinics, and tribal centers over 3 to 4 months. Tailored messages resonated with cultural values, while common themes included family and faith. Preferred materials were fact sheets, pamphlets, posters, videos, and visual stories. Participant evaluations showed high satisfaction, comfort sharing opinions, and improved understanding of how to take care of one's health.

**Lessons Learned:**

The LEC method fosters collective responsibility between community members and researchers to collaboratively address health needs. To support implementation, we offer best practices for LEC application, and customizable materials and guidance tailored to community preferences. This flexible, adaptable approach may enhance effectiveness, relevance, and sustainability of public health efforts across diverse settings.

## Abstract (plain language summary)

**Plain Language Summary: **An adapted Boot Camp Translation method was used to create culturally relevant health messages and materials for diverse communities. The process involved listening, empowering, and co-creating over 3 to 4 months. Participants engaged in familiar settings, resulting in tailored materials focusing on messaging that resonates with community perspectives and preferences. This approach improved understanding of health issues and fostered collaboration between researchers and communities.

Every community has its own unique context shaped by cultural, social, linguistic, environmental, and economic factors that can significantly impact the health and well-being of its members (National Academies of Sciences et al., 2017). These determinants of health often manifest in disparities, with underserved communities disproportionately burdened by poor health outcomes (Alcaraz et al., 2020; Havranek et al., 2015; National Academies of Sciences et al., 2017). This is particularly evident in cancer disparities, where certain racial, ethnic, and socioeconomic groups face higher cancer incidence and mortality rates (Alcaraz et al., 2020; National Cancer Institute, 2025; Zavala et al., 2021). Despite an overall decrease in cancer death rates since 1991, significant disparities persist among racial and ethnic groups in the United States. Native American populations experience cancer mortality rates 2 to 3 times higher than White individuals for several cancer types. Alaska Native populations have the highest colorectal cancer rates (CRC) globally (American Cancer Society, 2025). African American and Black populations face 40% to 50% higher mortality rates for various cancers compared to White individuals (American Cancer Society, 2025). Hispanic and Latino/a/e individuals, while having lower CRC incidence, are often diagnosed at later stages and face barriers to screening, with only 52% up to date on CRC screening (American Cancer Society, 2024).

These disparities highlight the urgent need for targeted interventions to address inequalities in cancer outcomes across diverse populations. Research has shown that a lack of culturally appropriate health messages contributes to disparities in cancer screening, especially among underserved populations. For instance, despite overall declines in invasive CRC rates, incidence has increased among Hispanic and Latino/a/e populations, particularly men, highlighting the need for tailored messages addressing specific barriers within communities (Garcia et al., 2018).

Limited health literacy compounds these challenges, as individuals may struggle to understand cancer prevention messages, navigate the health care system, and adhere to screening recommendations (Davis et al., 2002; Fleary et al., 2019). Engaging directly with community members allows public health practitioners to tailor messaging to specific needs, ensuring appropriate health literacy levels and honoring cultural values and belief systems. Recent systematic reviews have reinforced the importance of culturally competent and context-specific interventions in improving cancer screening behaviors among underserved populations (Liu et al., 2023). Such collaborative approaches help shape health initiatives that are culturally relevant, accepted, and actionable, thereby increasing their potential for success and sustainability (Kale et al., 2023; Wallerstein et al., 2020).

Traditional top-down models of health promotion have often failed to resonate with diverse communities, resulting in suboptimal uptake and limited impact (Wallerstein & Duran, 2006). Likewise, such approaches often fail to address the diverse health literacy needs within communities, resulting in materials that are not easily understood or actionable by the intended audience. In contrast, community-based methods have been used effectively in various public health contexts, including chronic disease management, environmental health, and health disparities. By leveraging the unique insights and expertise of community members, such approaches foster a sense of ownership and empowerment, enhancing the cultural appropriateness and effectiveness of health interventions. Studies have shown that community engagement improves the relevance and implementation of health initiatives, as community members are viewed as co-creators rather than passive recipients (Wallerstein & Duran, 2010).

Our approach redefines the traditional research model by positioning community members as active co-creators rather than research participants. We start by identifying community health priorities and collaboratively developing health promotion materials that address these specific needs, ensuring that the community's expertise and voice are central to the process. A recent review by Kale et al. (2023) highlighted the positive role of community-engaged research in facilitating the translation of research into ways that are relevant and valuable to communities, which can potentially address cancer disparities. A notable outcome of respecting and integrating diverse cultural backgrounds is the increase in the likelihood of community acceptance. The authors also report that culturally tailored cancer education programs can facilitate proactive steps to improve health as individuals see themselves and their values reflected in health messages (Kale et al., 2023). Thus, integrating community voices is pivotal in the co-creation of health messages and materials to ensure responsiveness and effectiveness.

However, while some studies have demonstrated the effectiveness of tailoring messages to specific cultural groups (Lapinski et al., 2024), others have highlighted the challenges and mixed results of such interventions, underscoring the importance of rigorous evaluation (Castro et al., 2010). Specifically, the Boot Camp Translation (BCT) method, which our approach adapts, has shown promise in engaging communities to translate evidence-based information into locally relevant and actionable health messages (Norman et al., 2013).

Our approach builds on these foundations by going a step further—not only engaging community members in adapting messages but also educating them about the health topics themselves. This additional layer of empowerment and education ensures that community members are not merely involved in the development of health messages but are also informed experts capable of disseminating health information effectively to others. By making health messages clear, concise, and culturally relevant, we aim to bridge the health literacy gap and empower individuals to make informed decisions about their health.

This manuscript describes our team's experiences in adapting and implementing a community-based approach to develop culturally relevant health messages and materials for diverse communities across the US. We share best practices identified and lessons learned through this process, providing a practical guide for researchers and public health program leaders seeking to engage communities in addressing health disparities. We detail the steps our team has taken to develop health messages and materials for several diverse communities across the US. By highlighting our successes and challenges, we hope to empower others to implement similar approaches and foster more equitable health outcomes in their own communities.

## Brief Description of Policy, Program, or Activity

Our research team refined an adapted version BCT, a community-based approach, to develop culturally and locally relevant messaging and materials. This method has been applied in diverse communities across the US, including Hispanic and Latino/a/e populations, African Methodist Episcopal church members and, most recently, American Indian and Alaska Native communities throughout the Great Plains and Pacific Northwest (Gautom et al., 2023; Norman et al., 2013; Thompson et al., 2019; Thompson et al., 2024).

Each of these projects was part of larger studies supported by various funding mechanisms. The adapted BCT approach used in each project underwent review by the Kaiser Permanente Institutional Review Board and was determined to be non-research, falling under the umbrella of quality improvement. This classification helps address common barriers to community partnerships, such as structural racism and restrictive policies, by fostering authentic community participation (Cunningham-Erves et al., 2024). By seeking exemption from traditional research classification, our approach is seen as quality improvement and fosters authentic community participation to ensure that co-created messages and materials genuinely reflect community needs and perspectives.

BCT, originally developed by researchers at the Colorado High Plains Research Network, is a validated method for engaging community members in translating evidence-based health information into ideas, messages and materials that resonate with target community members (Norman et al., 2013). In its original format, the process incorporates a schedule of in-person meetings combined with short, focused group telephone calls, requiring about 20 to 25 hours of participant time over 4 to 18 months depending on the scope of the project and complexity of the health topic (Norman et al., 2013). Recognizing that the original BCT method is time-intensive, our adapted approach optimizes the process to best meet the needs of the participating community.

The original BCT process, while effective, encounters challenges such as scheduling conflicts and considerable time commitments, which can be barriers for individuals with busy lives, multiple jobs, or travel obligations. Multiple in-person meetings can pose difficulties for rural or underserved communities. To address these issues, our adapted approach reduces the number of in-person meetings, utilizes flexible communication methods, and minimizes participant time requirements. This makes the process more accessible and manageable for a wider range of communities. Additionally, our approach is designed for real-world implementation in settings like community health centers or public health programs, which often face constraints related to staff and budget. By streamlining the process, we aim to facilitate participation in programs that prioritize immediate, actionable health initiatives while accommodating limited time and funding.

Our adapted BCT community engagement approach focuses on three core themes: 1) Listen, 2) Empower, and 3) Co-Create, or LEC. The LEC process helps community leaders and champions learn from community members about barriers and gaps in health care (listen), share health information in a collaborative way (empower), and develop messages and materials that resonate with and motivate community members to take control of their own health (co-create). The approach honors the local and cultural aspects of the community within the context of evidence-based health care and community-driven goals. Implementation of these three core themes are described below.

### Listen

Listening to the community is a critical part of the engagement. Learnings from community members, including patients, help inform an understanding of the gaps (e.g., access to resources, knowledge, screening adherence) and the cultural values and needs of the community. Understanding the health issue and concerns of a community helps identify the needs of the target population and create effective screening messages, leading to a more successful community engagement approach.

### Empower

Expert presentations are an integral element of the community engagement process as they engage and empower participants to become experts on a health topic and ultimately a voice for their community (Norman et al., 2013). Communities that are medically underserved may experience systemic barriers or face historical trauma that prevent them from fully engaging in healthful behaviors or accessing care. LEC acknowledges the important role of knowledge and having control over one's own health (Wallerstein & Bernstein, 1988). Participants help create messages and materials that can be shared across generations to meet an array of needs and foster self-empowerment. This sharing of knowledge is not meant to be didactic but rather an interactive exchange that builds confidence in community members and empowers individuals to share ideas and insights about health messaging that would be meaningful to their community. Along with community champions and leaders, we identify expert presenters (who may be from the local sector but could also be a regional or national expert) to share about the health topic of interest, local barriers and resources, and cultural values such as faith, tradition, storytelling, holistic health, and more. We also consider a culturally familiar and trusted environment where this community work should take place in an effort for participants to be their authentic selves, which enhances participant willingness to engage and speak freely.

### Co-Create

Community-based health interventions that are developed in close partnerships with a target community have positive impacts on health outcomes (Cyril et al., 2015; O'Mara-Eves et al., 2015). By inviting community members to actively participate in the creation of educational messages and materials, we move beyond generic, onesize-fits-all solutions and instead craft tailored resources that authentically reflect the community's language, cultural norms, and lived experiences. When community members are empowered as co-creators, they develop a profound sense of ownership over the final products. This fosters trust, buy-in, and long-term sustainability, as the community sees themselves reflected in the very tools designed to support their health and wellbeing. Ultimately, co-creation is not just a best practice—it is a necessity for developing impactful, community-driven initiatives that can serve as a catalyst for lasting change (Vaughn & Jacquez, 2020; Wallerstein et al., 2020).

## Implementation

**Figure 1.**
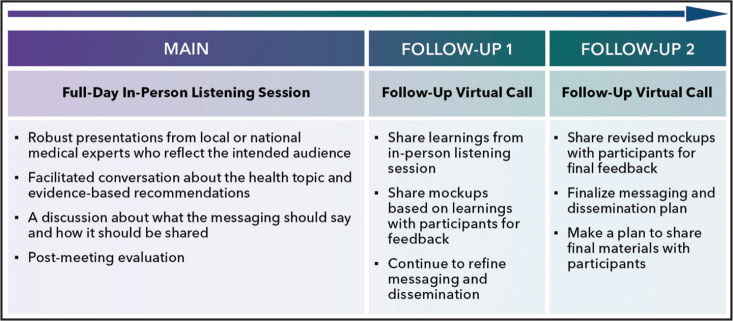
Listen, empower, and co-create (LEC) (adapted Boot Camp Translation [BCT]) format. From the research team website (in the public domain; permission is not required): http://bit.ly/41kUJc2.

LEC follows a format that includes a 5- to 6-hour in-person session, followed by two 60-minute follow-up virtual calls over a 3-month period (**Figure 1**).

The in-person session includes expert presentations on the relevant health topic, brainstorming sessions, and small group facilitated discussions. The presentations translate scientific and evidence-based recommendations and guidelines into language that is accessible to patients and community members. The presentations create a base of common knowledge and language that can be used throughout the sessions. The in-person session is interactive with many points for engagement and brainstorming opportunities. The group discusses the key concepts from the presentation that should be communicated (the contents of the message) and a diverse array of approaches to connect with the community (how to disseminate the message). Follow-up virtual meetings are intended for the research team to share drafted messages and materials created based on the in-person learnings with community members. These follow-up touchpoints provide an opportunity to gather feedback on the messages and materials, ensure accuracy, and maintain active engagement in the co-creation process. The collaborative approach enhances relevance and fosters a sense of shared ownership.

Successful implementation of this method requires a skilled project team. Roles include expert presenters, project coordinator, facilitators, and notetakers (**Table 1**). Expert presenters will share information about the health topic, including screening options, local barriers, and resources, and how the topic interfaces with the community. The content coordinator is responsible for managing the project timeline, developing the presentations on the health topic, and synthesizing insights gathered from the session into messages and materials. Additionally, the project coordinator is responsible for planning and executing the logistical aspects of the session. The recruiter will invite participants who represent the target community, ensuring a sample that can address the unique questions that need to be answered during the session. Reminder calls are made to ensure session attendance and engagement. Lastly, facilitators lead the discussion sections and breakout groups during the session and notetakers document the proceedings of the session, including general notes and detailed records from small group discussions. This approach not only facilitates the dissemination of crucial health information but also promotes community engagement through culturally appropriate practices.

**Table 1:**
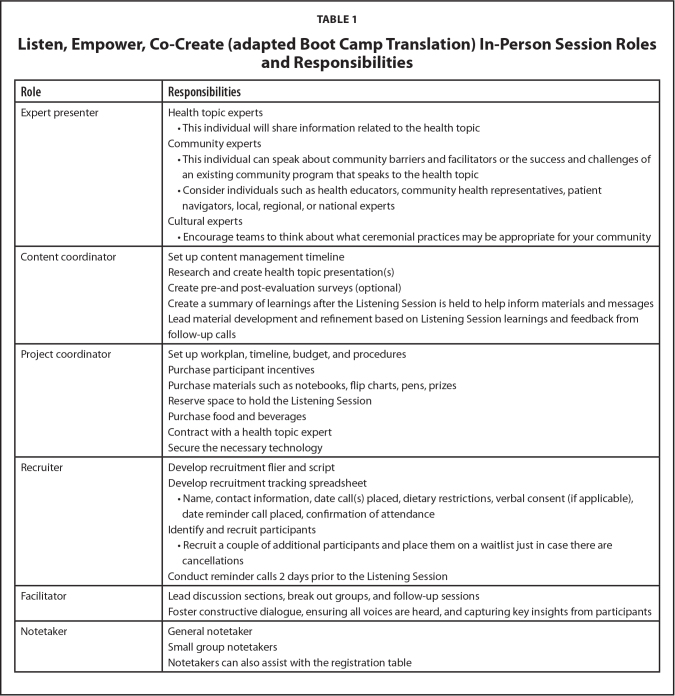
Listen, Empower, Co-Create (adapted Boot Camp Translation) In-Person Session Roles and Responsibilities

**Role**	**Responsibilities**

Expert presenter	Health topic experts
	•This individual will share information related to the health topic
	Community experts This individual can speak about community barriers and facilitators or the success and challenges of an existing community program that speaks to the health topic Consider individuals such as health educators, community health representatives, patient navigators, local, regional, or national experts
	Cultural experts
	•Encourage teams to think about what ceremonial practices may be appropriate for your community

Content coordinator	Set up content management timeline
	Research and create health topic presentation(s)
	Create pre-and post-evaluation surveys (optional)
	Create a summary of learnings after the Listening Session is held to help inform materials and messages
	Lead material development and refinement based on Listening Session learnings and feedback from follow-up calls

Project coordinator	Set up workplan, timeline, budget, and procedures
	Purchase participant incentives
	Purchase materials such as notebooks, flip charts, pens, prizes
	Reserve space to hold the Listening Session
	Purchase food and beverages
	Contract with a health topic expert
	Secure the necessary technology

Recruiter	Develop recruitment flier and script
	Develop recruitment tracking spreadsheet
	•Name, contact information, date call(s) placed, dietary restrictions, verbal consent (if applicable), date reminder call placed, confirmation of attendance
	Identify and recruit participants
	•Recruit a couple of additional participants and place them on a waitlist just in case there are cancellations
	Conduct reminder calls 2 days prior to the Listening Session

Facilitator	Lead discussion sections, break out groups, and follow-up sessions
	Foster constructive dialogue, ensuring all voices are heard, and capturing key insights from participants

Notetaker	General notetaker
	Small group notetakers
	Notetakers can also assist with the registration table

Over the last 7 years, our research team has implemented the LEC approach across different projects. Each initiative has engaged diverse populations, including Hispanic and Latino/a/e, Black and African American, and American Indian community members. The selection of communities for each project was guided by specific objectives from respective funding agencies. Participant recruitment involved convenience sampling via community partners and random sampling of eligible patients in clinical settings. LEC sessions were conducted in a variety of locations (i.e., clinics, churches, and tribal community settings) based on community member preferences. **Table 2** shows details about location, timing, and key questions.

**Table 2:**
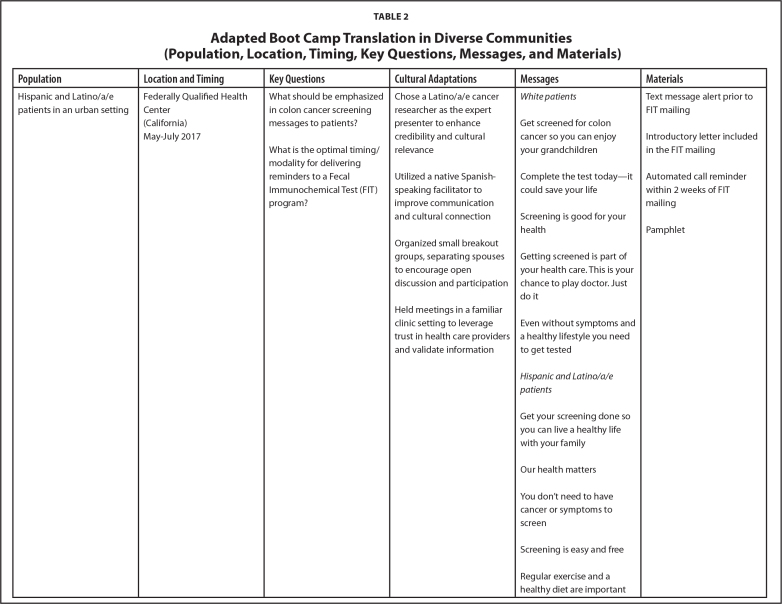
Adapted Boot Camp Translation in Diverse Communities (Population, Location, Timing, Key Questions, Messages, and Materials)

**Population**	**Location and Timing**	**Key Questions**	**Cultural Adaptations**	**Messages**	**Materials**

Hispanic and Latino/a/e patients in an urban setting	Federally Qualified Health Center	What should be emphasized in colon cancer screening messages to patients?	Chose a Latino/a/e cancer researcher as the expert presenter to enhance credibility and cultural relevance	*White patients*	Text message alert prior to FIT mailing
				Get screened for colon cancer so you can enjoy your grandchildren	
	(California)				Introductory letter included in the FIT mailing
	May–July 2017	What is the optimal timing/modality for delivering reminders to a Fecal			
			Utilized a native Spanish- speaking facilitator to improve communication and cultural connection	Complete the test today—it could save your life	Automated call reminder within 2 weeks of FIT mailing
		Immunochemical Test (FIT) program?			
				Screening is good for your health	
					Pamphlet
			Organized small breakout groups, separating spouses to encourage open discussion and participation	Getting screened is part of your health care. This is your chance to play doctor. Just do it	
			Held meetings in a familiar clinic setting to leverage trust in health care providers and validate information	Even without symptoms and a healthy lifestyle you need to get tested	
				*Hispanic and Latino/a/e patients*	
				Get your screening done so you can live a healthy life with your family	
				Our health matters	
				You don't need to have cancer or symptoms to screen	
				Screening is easy and free	
				Regular exercise and a healthy diet are important

**Table:**
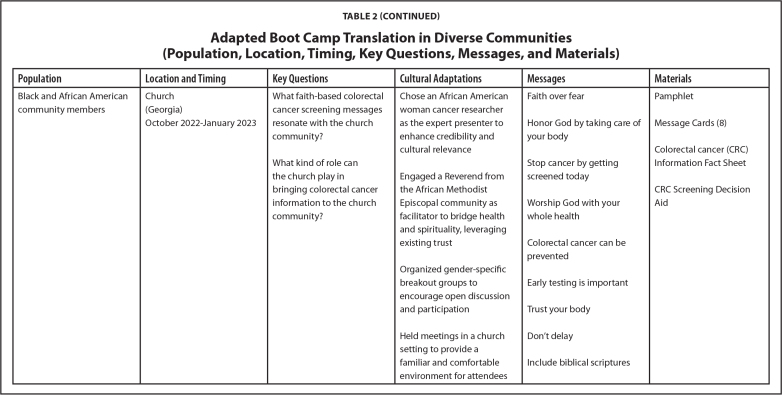


**Population**	**Location and Timing**	**Key Questions**	**Cultural Adaptations**	**Messages**	**Materials**

Black and African American community members	Church	What faith-based colorectal cancer screening messages resonate with the church community?	Chose an African American woman cancer researcher as the expert presenter to enhance credibility and cultural relevance	Faith over fear	Pamphlet
	(Georgia)				
	October 2022–January 2023			Honor God by taking care of your body	Message Cards (8)
					Colorectal cancer (CRC) Information Fact Sheet
		What kind of role can the church play in bringing colorectal cancer information to the church community?	Engaged a Reverend from the African Methodist Episcopal community as facilitator to bridge health and spirituality, leveraging existing trust	Stop cancer by getting screened today	
					CRC Screening Decision Aid
				Worship God with your whole health	
				Colorectal cancer can be prevented	
			Organized gender-specific breakout groups to encourage open discussion and participation		
				Early testing is important	
				Trust your body	
			Held meetings in a church setting to provide a familiar and comfortable environment for attendees	Don't delay	
				Include biblical scriptures

**Table:**
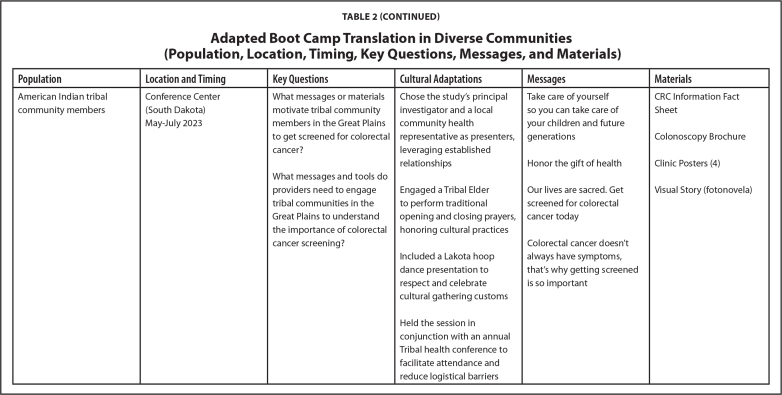


**Population**	**Location and Timing**	**Key Questions**	**Cultural Adaptations**	**Messages**	**Materials**

American Indian tribal community members	Conference Center (South Dakota)	What messages or materials motivate tribal community members in the Great Plains to get screened for colorectal cancer?	Chose the study's principal investigator and a local community health representative as presenters, leveraging established relationships	Take care of yourself so you can take care of your children and future generations	CRC Information Fact Sheet
	May–July 2023				
					Colonoscopy Brochure
				Honor the gift of health	Clinic Posters (4)
		What messages and tools do providers need to engage tribal communities in the Great Plains to understand the importance of colorectal cancer screening?	Engaged a Tribal Elder to perform traditional opening and closing prayers, honoring cultural practices		
				Our lives are sacred. Get screened for colorectal cancer today	Visual Story (fotonovela)
			Included a Lakota hoop dance presentation to respect and celebrate cultural gathering customs	Colorectal cancer doesn't always have symptoms, that's why getting screened is so important	
			Held the session in conjunction with an annual Tribal health conference to facilitate attendance and reduce logistical barriers

**Table:**
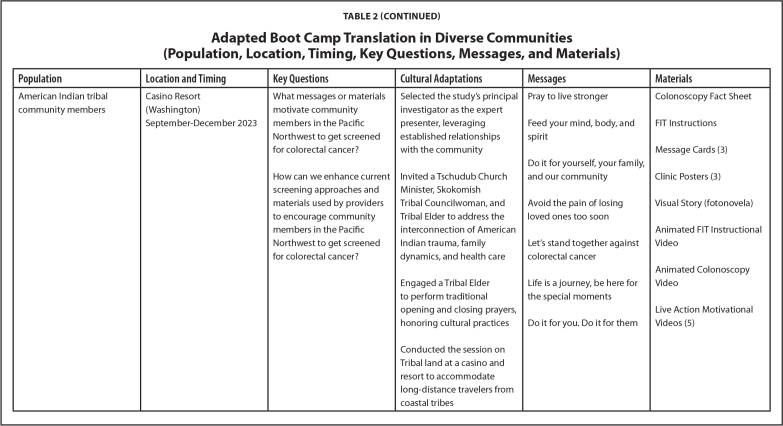


**Population**	**Location and Timing**	**Key Questions**	**Cultural Adaptations**	**Messages**	**Materials**

American Indian tribal community members	Casino Resort (Washington)	What messages or materials motivate community members in the Pacific Northwest to get screened for colorectal cancer?	Selected the study's principal investigator as the expert presenter, leveraging established relationships with the community	Pray to live stronger	Colonoscopy Fact Sheet
	September–December 2023			Feed your mind, body, and spirit	FIT Instructions
					Message Cards (3)
				Do it for yourself, your family, and our community	
		How can we enhance current screening approaches and materials used by providers to encourage community members in the Pacific Northwest to get screened for colorectal cancer?	Invited a Tschudub Church Minister, Skokomish Tribal Councilwoman, and Tribal Elder to address the interconnection of American Indian trauma, family dynamics, and health care	Avoid the pain of losing loved ones too soon	Clinic Posters (3)
					Visual Story (fotonovela)
				Let's stand together against colorectal cancer	Animated FIT Instructional Video
			Engaged a Tribal Elder to perform traditional opening and closing prayers, honoring cultural practices	Life is a journey, be here for the special moments	Animated Colonoscopy Video
					Live Action Motivational Videos (5)
				Do it for you. Do it for them	
			Conducted the session on Tribal land at a casino and resort to accommodate long-distance travelers from coastal tribes		

## Results

We successfully conducted the LEC approach in a variety of communities. The engagement took place in preferred venues for each community including churches, clinics, and tribal centers. Each project was conducted over 3 to 4 months in the last few years. Cultural adaptations were made for each project and are detailed in **Table 2**. Tailored messages and material were developed for each community, aligning with their unique values and preferences to enhance health literacy and comprehension. (**Table 2**). Common themes included integrating stories and messaging using a multigenerational or familial angle, highlighting local cancer statistics, emphasizing the importance of timely and regular screening in simple terminology, and including clear steps in the screening process. Preferred materials included fact sheets, pamphlets, message cards, in-clinic posters, visual stories (fotonovelas), instructional videos, and motivational videos.

Finally, to learn more about participant satisfaction, we distributed self-administered evaluations at the end of the in-person sessions. The evaluations used a paper survey format, and questions were presented using Likert scale structures and open-text comment sections. On average, participants reported (1) high levels of satisfaction with the engagement experience, (2) strong levels of comfort sharing their opinions on screening during the session, and (3) strong agreement that the session helped improve their understanding of health information, screening options, and the importance of developing the right messages and methods for sharing health information. Future evaluation opportunities include using learner verification and revision to further evaluate the suitability of materials and identify potential refinement needs for some of the products described in **Table 2** and posted at www.KPCHR-Engage.org (Chavarria et al., 2021).

## Lessons Learned, Implications, Recommendations to Others

### Best Practices for Implementing the LEC Approach

Through years of conducting LEC with diverse communities across the nation, we have developed a set of best practices for identifying effective expert presenters and participants, as well as selecting optimal session locations. These best practices highlight the critical importance of addressing tailored messaging and health literacy needs throughout every stage of the intervention—from selecting expert presenters who can effectively engage audiences to designing culturally relevant and accessible health materials.

***Identifying expert presenters.*** Collaborating with partner organizations is crucial in identifying expert presenters who can effectively communicate health information. These presenters should cover key topics such as the demographics of affected populations, detailed explanations of the health topic, various screening and detection methods, the importance of regular health screenings, local health disparity data, barriers to screening, and available resources. Additionally, they should discuss how to integrate cultural and local values into preventive behaviors. Typically, one or two expert presenters participate in each in-person session, focusing on their specific areas of expertise. Potential presenters may include local, regional, or national researchers, local health professionals, or community health representatives. A primary goal of these sessions is to create a comfortable environment that respects community and cultural practices. To achieve this, presenters and facilitators should align their communication style with community norms by using accessible terminology, incorporating cultural nuances, and matching the tone of the community. By doing so, these sessions can more effectively engage participants and convey crucial health information.

***Identifying and recruiting participants.*** After determining the key questions for the sessions, it is essential to identify the most suitable participants. While populations are often broadly defined using general descriptors like race and sex, a more nuanced approach to participant selection can yield richer, more targeted results. To achieve this, consider refining your recruitment criteria to focus on specific subgroups. Key characteristics to consider may include age range, primary language, level of acculturation, religious affiliation, geographic location, socioeconomic status, occupation, educational background, and relevant health behaviors or risk factors. By carefully tailoring your participant selection, you can ensure that the insights gathered are more precisely aligned with your research objectives and the specific health concerns of the community you are studying.

To foster active engagement of all participants throughout the sessions, the ideal number of community participants is between 12 and 16 individuals. This recommended group size allows for active participation throughout the sessions, as some individuals may be hesitant to share their experiences in larger settings. During the in-person session, smaller breakout groups of 6 to 8 participants are formed to gather reactions, preferences, and ideas regarding potential messages and materials. This approach creates a comfortable discussion environment for participants while maintaining a manageable number for the facilitator and research team to foster engagement effectively. To account for potential cancellations or no-shows, the study team should over-recruit, aiming to enroll 16 to 18 individuals. This strategy helps ensure that even if some participants are unable to attend, the final group size remains within the ideal range for productive discussions and meaningful interactions.

To effectively recruit participants, collaborate closely with partner community organizations to identify optimal recruitment methods that place the message in a familiar context. For instance, when targeting older, monolingual Hispanic and Latino/a/e individuals, consider placing flyers or hosting tabling events at a Mercado Latino (Latino Market). If your target population is defined more by behavioral characteristics such as health and lifestyle choices, look to existing health groups like nutritional or diabetes programs for potential participants. Recruitment strategies may include personalized letters emphasizing the value of participants' experiences and insights, concise flyers distributed via mail or email through community, religious, health system, or recreational groups, or one-on-one recruitment phone calls from trusted sources within the partnering organization. By tailoring your approach to the specific demographic and leveraging existing community networks, you can increase the likelihood of reaching and engaging your target participants effectively.

***Location.*** Choosing the right venue is crucial for successful sessions. Collaborate with the community to select a safe, accessible location that encourages open dialogue and shared power. Previous sessions have been held in community clinics, church halls, and tribal centers. Ensure the space is intimate enough for discussion, accommodates refreshments, and considers virtual accessibility. Create an inviting atmosphere by incorporating local cultural practices, such as traditional prayers, presentations, and food. This approach fosters a respectful environment, enhancing participant engagement and session success.

***Selecting a date.*** Selecting an optimal date and time is also crucial for maximizing session attendance. Collaborate closely with the partner community organization to identify any potential conflicts, such as popular local events, cultural observances, or holidays that might hinder participation. Consider aligning the in-person session with an existing community event or meeting, as this strategy can streamline recruitment and accommodate individuals from various parts of the community who may have already arranged time off from work or personal obligations. By thoughtfully scheduling the session, you can reduce barriers to attendance and increase the likelihood of diverse community representation. This approach not only respects participants' time and commitments but also demonstrates cultural sensitivity and community awareness, further enhancing the session's potential for success.

## Conclusion

The LEC method fosters a sense of shared responsibility between community members and researchers, empowering them to collaboratively address the unique health needs and challenges of specific populations. By bridging the gap between research and real-world application, LEC integrates communities into the research process to ensure interventions are culturally appropriate, relevant, and rooted in trust between academic partners and the community (Haldane et al., 2019). Its versatility makes it adaptable to a wide range of health topics, ensuring its broad applicability.

The flexibility of the LEC method is central to its success, as it allows for tailoring to the specific needs of each population, ensuring relevance, resonance, and long-term sustainability. This community-driven approach is vital for reducing health disparities, promoting health equity, and protecting the well-being of diverse populations. By building trust and credibility—essential components of effective public health outreach—the LEC method lays the groundwork for impactful and lasting interventions.

However, while LEC shows great promise in developing culturally tailored health messages, it is not without limitations. The qualitative nature of its data and its focus on specific local communities limit the generalizability of findings. Additionally, reliance on self-reported satisfaction data collected immediately after sessions offers valuable insights but does not capture long-term impacts on knowledge, attitudes, or behavior change. Logistical challenges such as scheduling and time commitments may also hinder participation from some community members. Future research should incorporate quantitative measures to evaluate the effectiveness of co-created materials on health outcomes, assess adherence to health literacy standards, and use validated tools to measure readability, understandability, and actionability.

Recognizing that not all communities have the resources to undertake this type of engagement work, we provide customizable materials on our research website (www.KPCHR-Engage.org) along with guidance for implementing this approach in resource-constrained settings. Our implementation guides offer tailored strategies for conducting this work effectively while addressing the unique needs and opportunities within diverse communities.

In conclusion, the LEC community engagement approach enhances the effectiveness, cultural relevance, and sustainability of public health initiatives across a broad range of populations. By fostering collaboration and empowering communities as equal partners in public health efforts, LEC serves as a cornerstone for meaningful progress in reducing health disparities and promoting equity. We encourage public health practitioners and leaders to adopt this community-centric model as a transformative tool for driving impactful public health advancements.
